# Human Mesenchymal Stromal Cell Sheet Enhances Allograft Repair in a Mouse Model

**DOI:** 10.1038/s41598-017-08804-2

**Published:** 2017-08-11

**Authors:** Xifu Shang, Bing Shu, Yongjun Wang, Zhengliang Luo, Guangxi Wang, Shane Barton, Massimo Max Morandi, Christopher Kevil, Yufeng Dong

**Affiliations:** 1 Department of Orthopedic Surgery, Anhui Provincial Hospital, Hefei, Anhui China; 2 Longhua Hospital, Shanghai University of Traditional Chinese Medicine, Shanghai, China; 3 Key Laboratory, Ministry of Education of China, Shanghai, China; 4 Rehabilitation School, Shanghai University of Traditional Chinese Medicine, Shanghai, China; 5 Department of Orthopedic Surgery, Louisiana State University Health Sciences Center, Shreveport, LA USA; 6 Department of Pathology, Louisiana State University Health Sciences Center, Shreveport, LA USA

## Abstract

To determine whether cell sheets generated with long-term passaged (P10) aging human mesenchymal stromal cells (MSCs) could be used for bone tissue regeneration as tissue engineered periosteum in a femoral allograft mouse model similar to fresh passaged (P3) young MSCs. At 3 weeks after transplantation of MSC sheets, results showed more bony callus formed between allograft and host bone ends in both young P3 MSC and aged P10 MSC sheet-wrapped groups when compared to allograft alone. At 6 weeks, while both MSC sheet-wrapped allografts showed more bony callus formation when compared to allograft alone groups, the bony callus size in aged P10 MSC sheet groups was significantly less than young P3 MSC sheet groups. Biomechanical testing confirmed that P3 MSC sheet-grafted femurs had the highest biomechanical strength in the three groups. Histology sections showed that the area of the chondriod callus in the aged P10 MSC sheet groups was significantly larger than in P3 MSC sheet groups. Finally, a significant increase of chondro-osteoclast activity was observed in the P3 MSC sheet-grafted femur. Our data demonstrates that extensive long-term culture-induced MSC aging impaired their osteogenic ability and subsequent bony callus formation, and could be used to induce cartilaginous callus formation.

## Introduction

Limb salvage procedures following massive segmental bone loss due to traumatic extremity injuries or skeletal tumor resections are a major challenge in the field of orthopedics^1, 2^. Large bone defect surgeries like these require devitalized segmental allograft transplantations to replace missing host bone segments; however, significant problems often arise due to the impaired ability of the devitalized allograft to incorporate into the host bone since lacking functional bone forming cells inside allograft^3, 4^. One potential treatment strategy entails isolating mesenchymal stromal cells (MSCs) from the patient, expanding them in culture to form a cell sheet, and wrapping cell sheets on devitalized allografts as a tissue-engineered periosteum prior to transplantation. Following transplantation, the MSCs are exposed to endogenous factors within the injured and healing region that promote their osteogenic differentiation, resulting in increased bone callus formation and enhanced osteointegration of the allograft and the adjacent patient bone^5, 6^. Due to the low frequency (0.01% to 0.001%) of MSC in total bone marrow cells, it is essential to culture and populate MSCs *in vitro* before putting them to therapeutic use^7^. However, *in vitro* culture has proven to be difficult since the telomere length shortens after each division cycle, leading to a gradual cell aging with an increased cellular senescence and a decreased culture life span^8, 9^. Thus, it is necessary to evaluate the regenerative capacity of long-term *ex vivo* expanded aging MSC for tissue regeneration.

We have demonstrated the therapeutic effect of early passaged young (P3) primary mouse MSCs following short-term cell sheet culture by maintaining their stromal cell characteristics (Oct4, Sox2, Nanog, and CD105 expression). Furthermore, we have identified the optimal cell number for generating appropriately sized cell sheets in 24 hours using mouse young MSCs^5^. To move this technology a step closer to clinical application, we need to replicate the therapeutic effect of cell sheets using human MSCs. Although our short-term cultured MSC sheets showed a significant increase of bone callus formation around allografts, studies on cell sheets generated with aging MSCs from prolonged *ex vivo* cell culture are still essential in developing ready-to-use cell sheets for clinical application. Additionally, prolonged MSC culture provides extra time for the surgeon to have a flexible transplantation schedule, which avoids any unnecessary MSC discarding.

Progressive loss of stem cell functionality caused by the reduction in stem cell number or perturbed cell-cycle activity has been reported in aged animals^10^. Depletion of the stem cell pool with age may occur because these cells lose self-renewal activity and terminally differentiate, thereby exiting the stem cell pool, or because they undergo apoptosis or senescence^11^. Similarly, when MSCs cultured *in vitro extensively*, reduced self-renewal and accelerated terminal differentiation, as well as enhanced apoptosis or senescence are often observed^12^. Therefore, studying whether extensively passaged aging P10 MSCs still maintain their therapeutic effects after a short-term cell sheet culture on thermo-responsive culture dishes, similar to P3 passaged young MSCs, is important for clinical settings.

## Results

### Extensive *ex vivo* culture induces MSC aging

**Figure 1:**
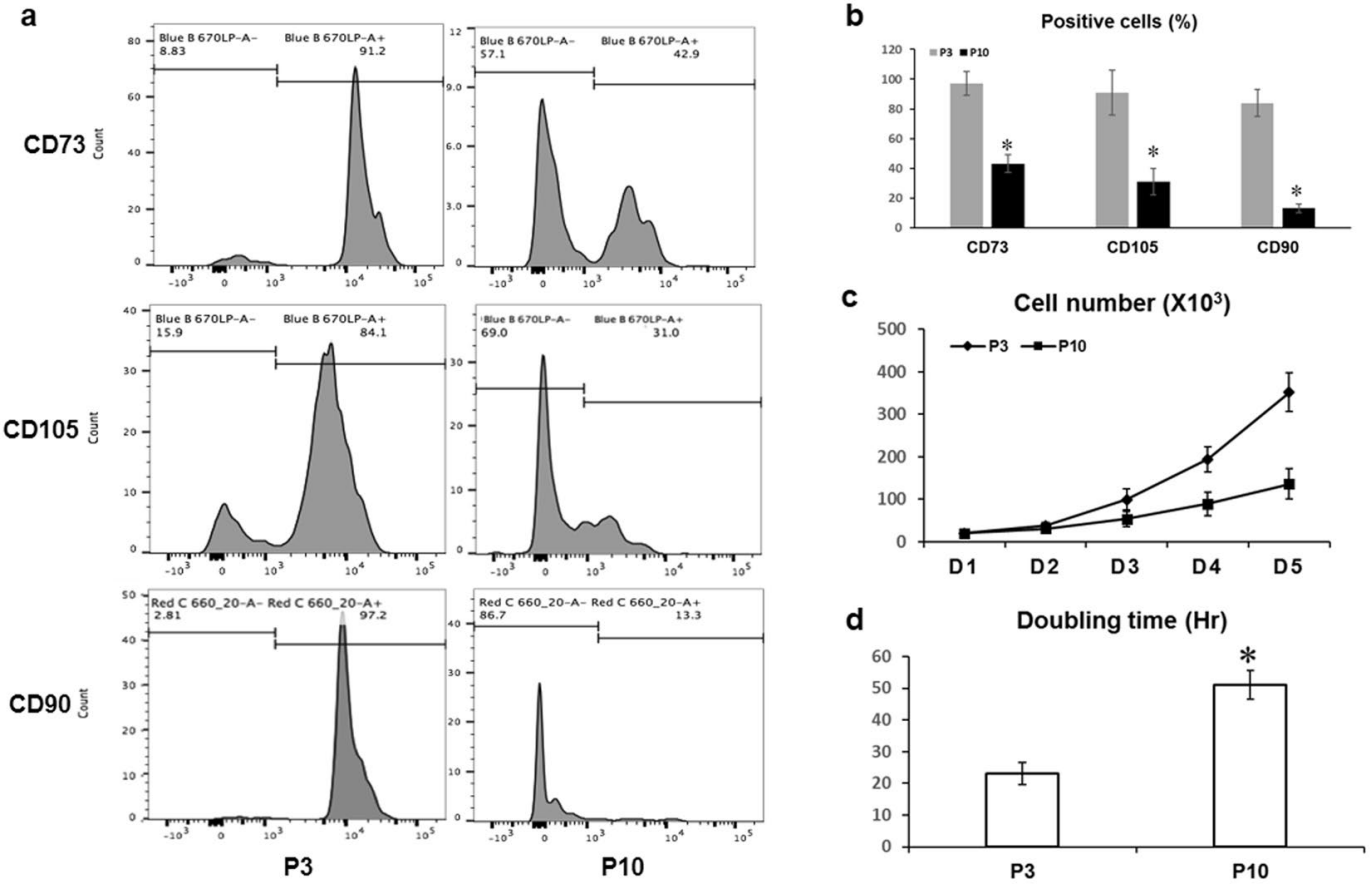
Long-term cell culture induces aging in MSCs. (**a**) Representative Flow Cytometry histograms showing CD73, CD105 and CD90 positive MSCs in cultured P3 and P10 MSCs. (**b**) Quantified percentage of CD73, CD105 and CD90 positive MSCs in P3 and P10 MSCs from 6 patients. (**c**) Representative growth curve of P3 and P10 MSCs from one donor. (**d**) Cumulative population doubling time (DT) of P3 and P10 MSCs is shown in hours (Hr). *P < 0.05.

To isolate functional stromal cells is important not only to study the molecular mechanisms but also for the establishment of stromal cell–based therapeutics. Here we adopted a protocol to isolate human bone marrow derived MSC using a plastic adherent method. Human bone marrow aspirates were obtained from six patients, and plastic-adherent fibroblast-like colonies were observed in all donor samples within the first 5 days of cultivation. Flow cytometry analyses (Fig. 1a) indicated that MSCs obtained from six separate preparations ranged from 84.5% to 97.8% positive for stromal cell markers CD105, CD73 and CD90 in P3 MSCs. However, in P10 MSCs, the positive population of CD105, CD73 and CD90 significantly reduced to 42–13%, suggesting a tendency for loss of stromal phenotype during long-term culture. To eliminate hematopoietic stromal cell contamination, hematopoietic marker CD34 was analyzed. Less than 0.5% of CD34 positive populations in newly isolated human MSCs were observed (data not shown) demonstrating that our MSCs were not contaminated with hematopoietic cells. Finally, the average percentage of positive CD105, CD73 and CD90 MSCs in P3 and P10 cultures from flow cytometry data was quantified and shown in Fig. 1b.

**Figure 2:**
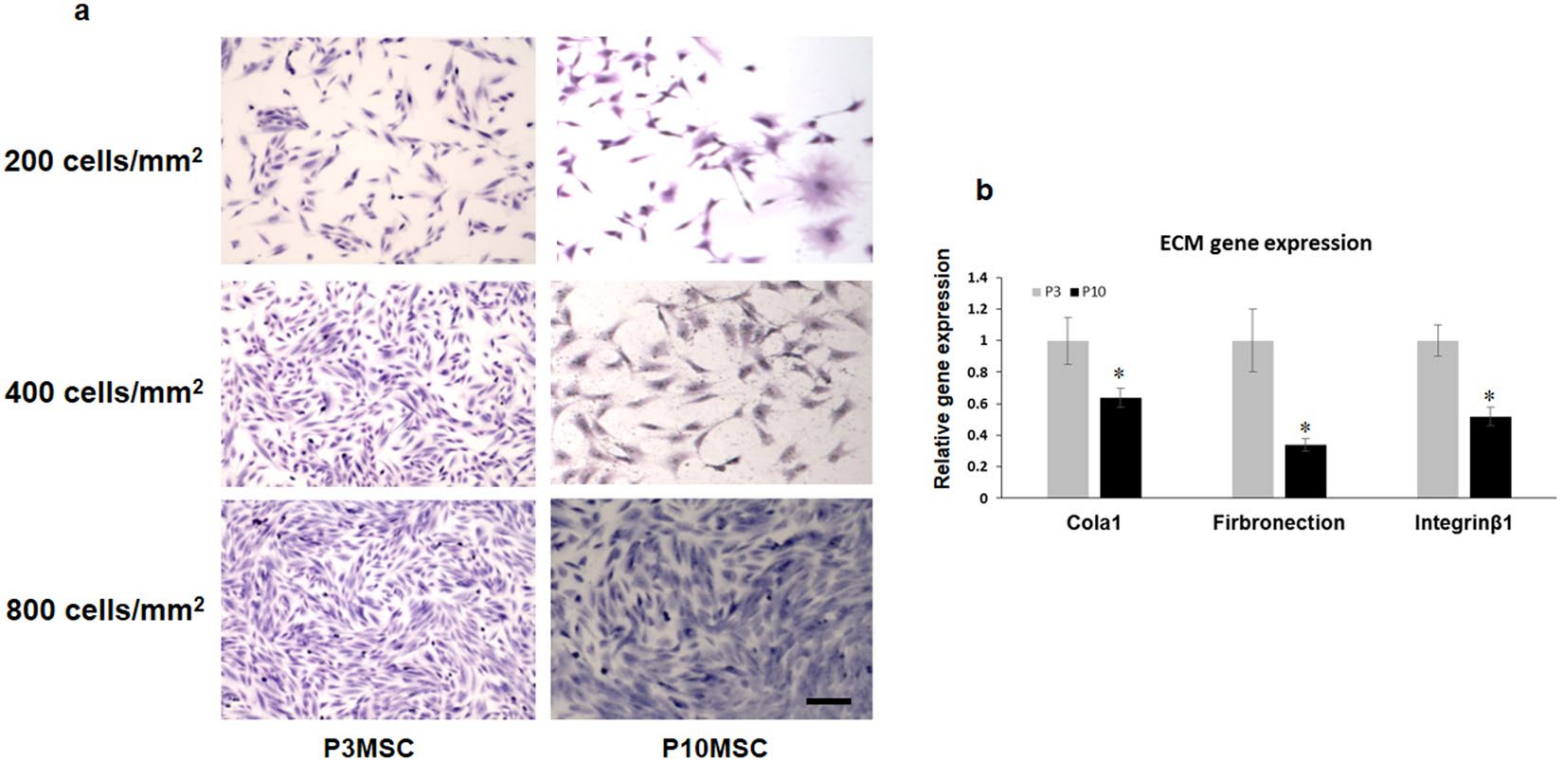
Cell morphology change during culture and generation of MSC sheets. (**a**) P3 and P10 MSCs were seeded at different densities for 24 hours and visualized by Giemsa staining. Scale bar, 100 um. (**b**) Expression of ECM related genes was tested by Real-time PCR using total RNA isolated from P3 and P10 MSC sheets, including the type 1 collagen (Cola1), fibronectin, and integrinβ1. *P < 0.05.

During MSC expansion, the proliferative rates of P3 and P10 cultures were monitored by growth curve and cell doubling time (DT). Initial 20.0 × 10^3^ MSCs were seeded to inoculate a 75 cm^2^ culture flask and harvested daily for up to 5 days before reaching confluence. As expected, the cumulative cell number in P3 MSCs was in general significantly higher than that in P10 MSC cultures, indicating a better proliferative activity in P3 MSCs (Fig. 1c). Consistent with this result, the DT mean value in P3 MSCs (26.1 ± 2.5 hours) was much shorter than in P10 MSCs (51.1 ± 4.2 hours), indicating that P3 MSCs required less time to duplicate when compared to P10 MSCs (Fig. 1d). To evaluate the cell morphology change during passaging, MSCs from P3 and P10 were stained by Giemsa solution. At an early phase of P3 culture, more than 90% of MSCs showed a “fibroblast-like” shape and usually formed an almost uniform cell monolayer at confluence (Fig. 2a, left panel). In P10 cultures, the cells varied in size and shape. Some cells became much larger with an irregular and flat shape, and nuclei became more circumscribed in phase contrast microscopy (Fig. 2a, right panel).

To further determine how many MSCs were needed to form a monolayer cell sheet in 24 hours, we seeded 3 different densities of P3 and P10 MSCs into 6-well plates as shown in Fig. 2a. Our results clearly showed that a monolayer cell sheet was formed in plates with seeding density of 400 and 800 cells/mm^2^ when P3 MSC was used. Meanwhile, seeding density of 800 cells/mm^2^ was required for P10 MSCs to form cell sheets in 24 hours due to its slower proliferation rate. We then decided to generate cell sheets for *in vivo* experiments using seeding density at 400 cells/mm^2^ for P3 MSCs and 800 cells/mm^2^ for P10 MSCs. To further show the difference of ECM in P3 and P10 MSC sheets, we also measured the expression of the ECM related genes using total RNA isolated from P3 and P10 MSC sheets, including the type 1 collagen (Cola1), fibronectin, and integrinβ1, that showed a significant decrease of expression in P10 sheets when compared to P3 sheets (Fig. 2b) suggesting a progress loss of ECM production during long-term culture.

### MSC sheets enhance allograft healing

**Figure 3:**
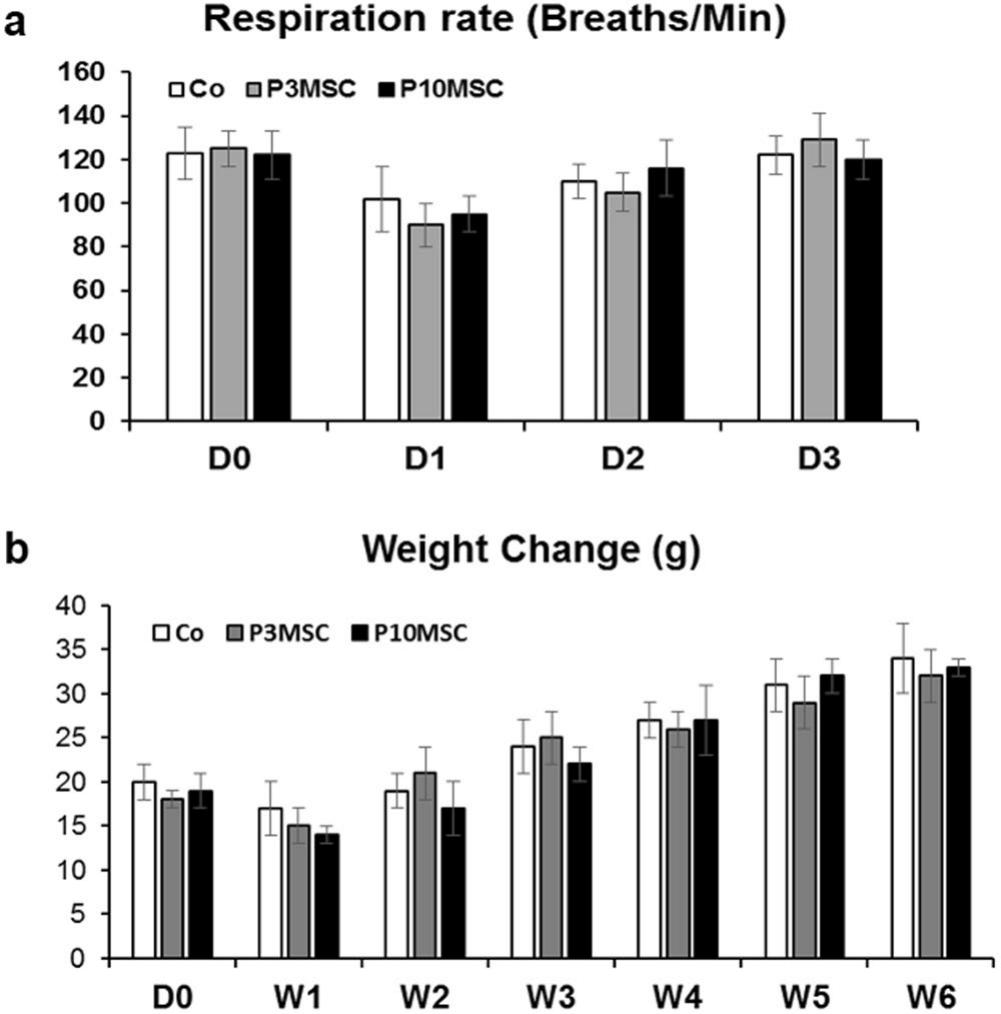
Gross observations before and after mouse allograft surgery. (**a**) Respiration rates were measured by visually counting the number of chest movements per minute (breaths/min) (n = 6) at day 0 (D0), day 1(D1), day 2(D2), and day 3(D3). (**b**) Values represent the weight change compared with the starting weight (D0) before surgery and after surgery at week 1 (W1), week 2 (W2), week 3 (W3), week 4 (W4), week 5 (W5), week 6 (W6).

Since our previous study showed short-term cell sheet culture does not affect MSC “stemness”^5^, we next transplanted human P3 and P10 MSC sheets into a C57/BL6 mouse femur bone allograft model as tissue-engineered periosteum^13^. Observation of the behavior of the animals is most important in the first 3 days of the experiment, since it is expected that rapid or delayed hyperacute transplant rejection may occur. Respiration rates were measured as baseline before allograft surgery (D0) and at 1, 2, and 3 days after surgery. Our observation data showed that in all mice, respiration rates measured within the normal range (100 and 200 breaths/min), and no significant difference was noticed between allograft alone and MSC sheet groups (Fig. 3a). Furthermore, among the three groups, no significant changes or differences were observed regarding infection, skin coloration (i.e. redness), posture or activity after surgery. Finally, animals in all three groups showed a slight loss of body weight at one week after surgery, followed by a recovery at 2, 3, 4, 5, and 6 weeks after surgery. The same kinetics within the three groups (Fig. 3b) indicate that human MSCs transplantation is well tolerated in this allograft mouse model.

**Figure 4:**
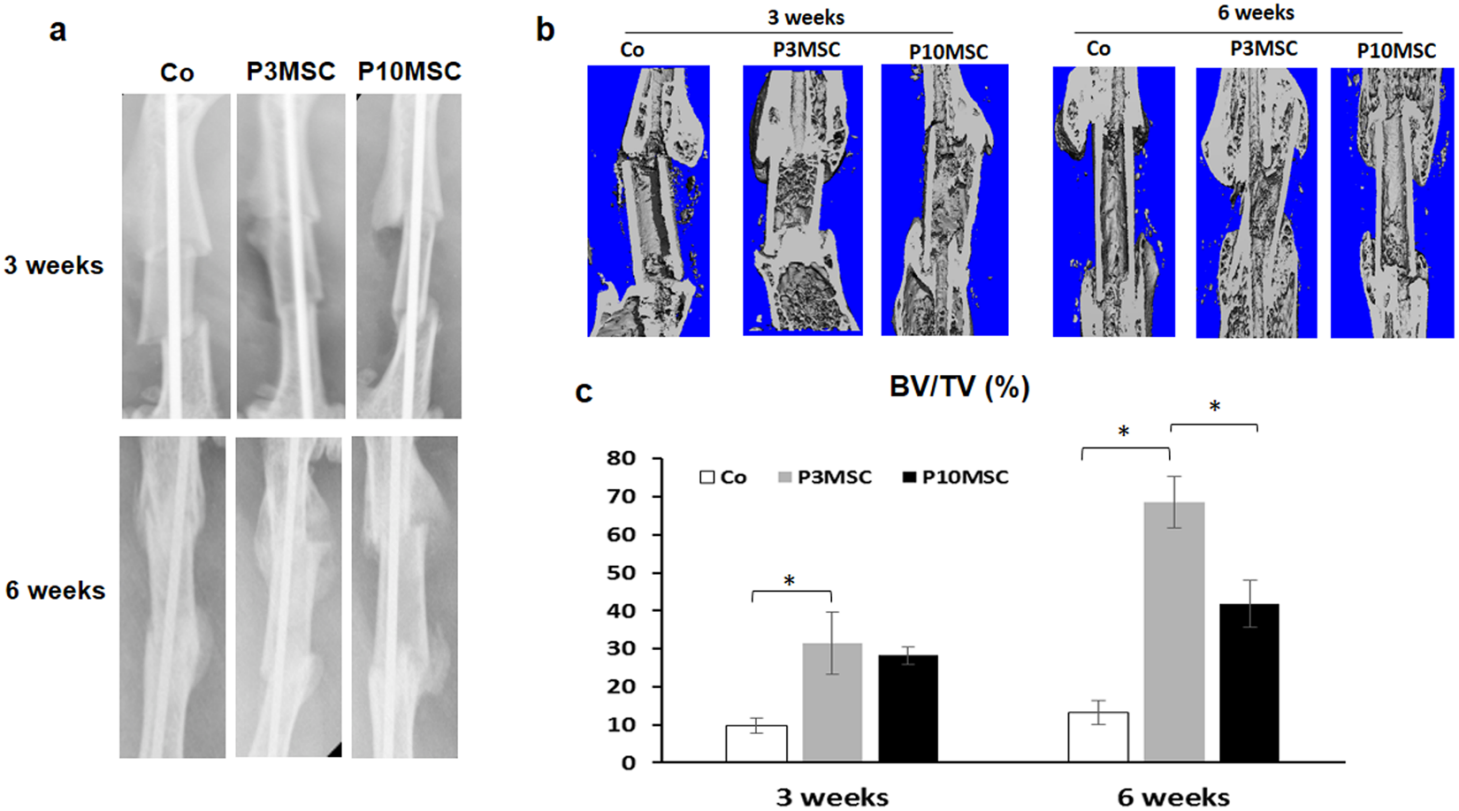
Enhanced callus formation in both MSC sheets wrapped allografts at 3 and 6 weeks post-surgery. (**a**) Representative X-ray images of allograft healing at 3 and 6 weeks post-surgery. (**b**) Representative M icro-CT images (cross-section) of allograft healing at 3 and 6 weeks post-surgery. (**c**) Quantification of the bone volume (BV) over total tissue volume (TV) shows a greater bony callus formation in the P3 MSC sheets groups in comparison to the P10 MSC sheets groups (n = 6). *P < 0.05.

To analyze the allograft healing process, we first monitored new bone callus formation using X-ray (Fig. 4a). At 3 weeks post-surgery, the fracture line between the host bone and allograft disappeared in both sheet-wrapped groups. However, in the control graft alone group, the fracture line remained at this time point, indicating more bony callus formed between allograft and host bone ends in both P3 and P10 MSC sheets wrapped allograft groups. At 6 weeks post-surgery, although a greater bony callus were observed in both MSC sheets groups when compared to allograft alone groups, the bony callus area in P10 MSC sheet groups was significantly less than that in P3 MSC sheet groups. To further validate our X-ray findings, we performed Micro-CT analysis to quantify the newly formed bony callus at 3 and 6 weeks post-surgery (Fig. 4b). Consistent with X-ray findings, the CT results clearly showed that the size of the newly formed bony callus in P3 MSC sheet groups was larger than that in the P10 MSC sheet and allograft alone groups. Although the percentage of BV to TV in P10 MSC sheet groups was less than in P3 MSC sheet groups, P10 MSC groups indeed showed a better callus formation when compared with allograft alone groups (Fig. 4c).

**Figure 5:**
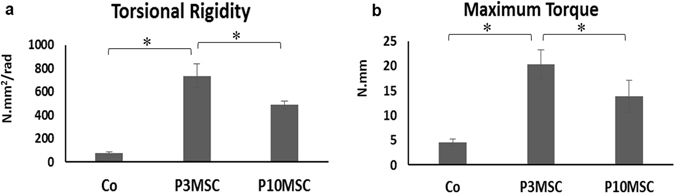
Enhanced biomechanical properties in MSC sheet-wrapped allografts at 6 weeks post-surgery. Mechanical properties of femur samples (n = 6) in groups of allograft alone, P3 MSC sheet/allograft, and P10 MSC sheet/allograft. (**a**) Torsional rigidity. (**b**) Maximum torque. *P < 0.05.

Since the success of bone repair should be defined by restoration of the biomechanical properties of the original bone^13^, we next performed torsional testing to measure the strength of the bone allograft samples at 6 weeks post-surgery. As would be expected from the known volume of bony callus in Micro-CT data, both P3 and P10 MSC sheet groups had significantly higher torsional properties than did the allograft alone groups. Torsional rigidity for P10 MSC-sheet groups (486.3 ± 68.1 N.mm^2^/rad) was 10-fold higher than in allograft alone groups (49.3 ± 7.4 N.mm^2^/rad), whereas the torsional stiffness for the P3 MSC sheet groups (735.6 ± 118.9 N.mm^2^/rad) was 15 times greater (Fig. 5a) than in allograft alone groups. Maximal torque analysis further showed that P3 MSC sheet groups had the highest value of peak torque in the three different allograft groups, confirming the best stability and integration with the native bone (Fig. 5b).

### Delayed bony callus calcification in aged MSC sheet-wrapped allograft healing

**Figure 6:**
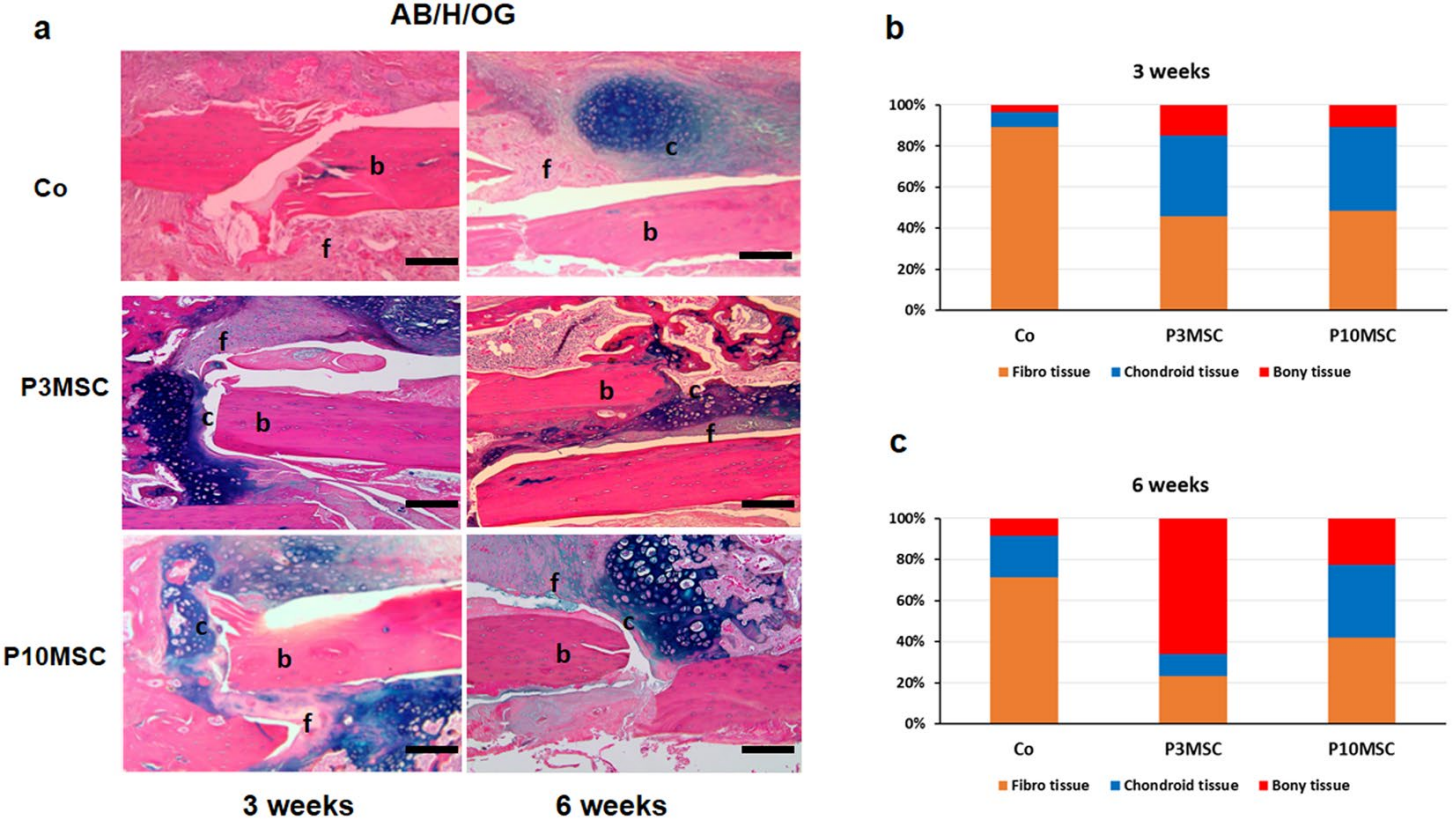
Enhanced chondrogenesis in aged MSC sheet wrapped allografts. (**a**) Representative images of callus between host bone and allograft in 3 groups. Histomorphometrical analysis of Alcian blue/Hema/Orange G (AB/H/OG) stained sections at 3 weeks (left) and 6 weeks (right) post-surgery. Specific regions are labeled as follows: b, bone; f, fibrous tissue; c, cartilage tissue. (**b**) Quantification of percentage of the bone, fibrous tissue, cartilage tissue in total callus at 3 weeks (n = 6). (**c**) Quantification of percentage of the bone, fibrous tissue, cartilage tissue in total callus at 6 weeks (n = 6). Scale bar, 400 um. *P < 0.05.

To study the histologic changes in callus, we performed AB/H/OG staining in samples from 3 and 6 weeks post-surgery. Sections from 3 weeks after surgery showed that larger chondroid tissues and hard callus were formed surrounding the gaps between allograft and host bone in both MSC sheet-wrapped allograft groups (Fig. 6a left) when compared with control allograft alone groups suggesting an enhanced chondro-osteogenesis in both cell sheet groups. At 6 weeks post-surgery, abundant chondroid tissues filling the site between host bone and allograft were still observed in the P10 MSC sheet group, while the size of the chondroid tissues was significantly smaller in the P3 MSC sheet groups. In contrast, the bony callus area in P3 MSC sheet groups was much larger than in P10 MSC sheet groups, indicating an accelerated endochondral ossification in P3 MSC sheet groups (Fig. 6a right). Histomorphometric analysis of histologic sections further confirmed that although both MSC sheet groups had a similar amount of chondro-osteoid callus at 3 weeks post-surgery (Fig. 6b), more rapid bony callus was formed in P3 MSC sheet groups when compared with P10 MSC sheet groups at 6 weeks post-surgery (Fig. 6c).

### Enhanced osteoclast activity in P3 MSC sheet-mediated bony callus formation

**Figure 7:**
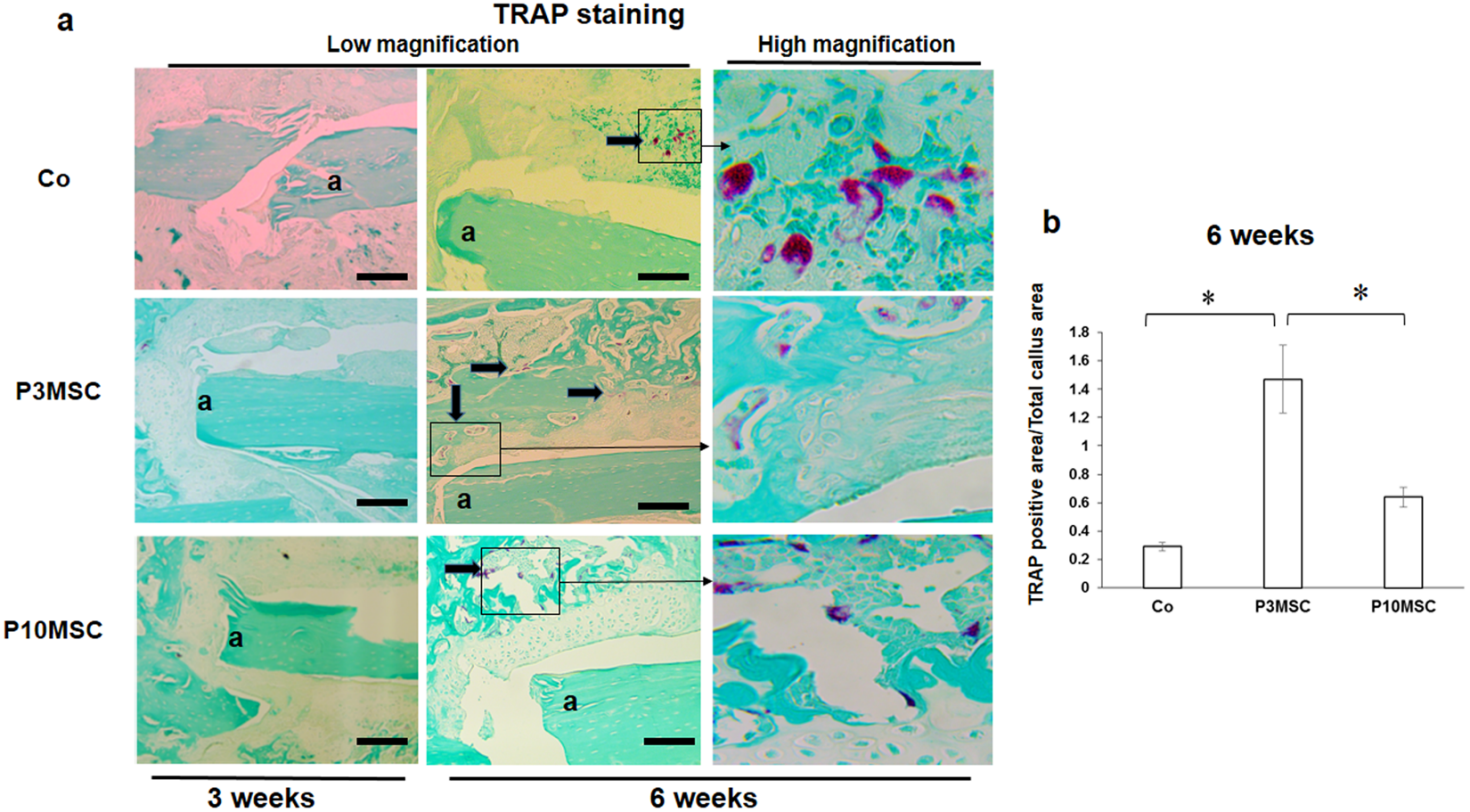
Enhanced osteoclast activity and callus remodeling in young P3 MSC sheet wrapped allografts. (**a**) Representative images of TRAP staining in new callus at 3 weeks (left) and 6 weeks (right) post-surgery with high magnification. (**b**) Quantification of the percent ratio of TRAP staining positive osteoclast area vs total callus area (n = 6) at 6 weeks post-surgery. Allograft is labeled as: a. Scale bar, 400 um. *P < 0.05.

Since the ossification of fibrocartilaginous callus requires organized osteoblast and osteoclast activity^14^, we performed TRAP staining to determine whether osteoclast is involved in this callus mineralization process. Trap staining results from sections at 3 weeks did not show significant staining or difference in newly formed callus among the three different groups (Fig. 7a, left). In contrast, TRAP staining showed an increased chondro-osteoclast activity in callus at 6 weeks post-cell sheet transplantation (Fig. 7a, right). Particularly, there were limited chondro-osteoclasts observed at small area in control callus, while more chondro-osteoclasts were widely distributed in large area of callus in P3 MSC sheet groups. When quantification analysis was performed with TRAP staining, we observed significantly more osteoclast activity in P3 MSC sheet groups compared to P10 MSC and allograft alone groups (Fig. 7b).

## Discussion

It is estimated that approximately 1.6 million bone grafts are used each year to regenerate bone that was lost due to trauma or disease^15^. Today, the “gold standard” of bone grafts is still an autograft. Unfortunately, the harvest of an autograft is not always possible and might lead to co-site morbidity and excessive pain^16^. Another option is the bone allograft, which is readily available for transplantation. These bone allografts have been preserved from deceased persons who have been carefully screened for diseases, including HIV and hepatitis infection. The host bones will first grow into the allograft, then replace the allograft with the host bone. Rejection of the grafted bone is rare because there are few fragments of the donor’s cells in the allograft to induce an immunoreaction^15^. The greatest problems with allograft reconstruction are the chance of graft fracture; and the failure of healing between the graft and the adjacent patient bone due to limited bone formation capability^17^. Therefore, there is a clear clinical need to develop revitalized allografts that are comparable or close to autografts to efficiently replace the missing bone tissue. To address this major clinical problem, our research team has developed a “tissue-engineered periosteum” composed of an MSC sheet that has been extensively tested in animal models. We have demonstrated that MSC sheets generated with fresh isolated cells possess enhanced potential for both chondrogenic and osteogenic differentiation^5^. Despite the clear-cut biologic advantages of MSC sheets for bone regeneration, several drawbacks still need to be addressed for their clinical use: 1) while three cell passages are adequate for generating an appropriate number of MSCs to create cell sheets for a much smaller mouse femoral allograft repair, many more passages will be required to generate the number of MSCs required for a human critical-sized bone defect; 2) the reduced proliferative capacity of aged MSCs may change the cell numbers required for seeding and generating MSC cell sheets of varying sizes; and 3) since extensive passaging of MSCs can lead to cell aging with reduced “stem-like” characteristics, we will need to define the precise effect of MSC aging on cell sheet-induced bone regeneration in our allograft model.

To generate high-quality MSCs for research and future clinical application, we first isolated human bone marrow MSC from 6 patients using a modified cell plastic adherent method. In accordance with the literature, immunophenotypic analysis of all MSC preparations confirmed that the detection level for positive markers was much higher in early passages at 3 compared to late passages at 10. Furthermore, some P10 MSCs significantly changed their initial morphology, and the cytoplasm began to be granular with increased cell size and reduced capability to reach a confluent monolayer suggesting an aging process in cells. Most of the P3 MSCs maintained their initial morphology, continuing to generate a very dense cell monolayer. These results support the notion that long-term culture has an incremental impact on “stemness” potential of MSCs.

Population doubling times were also recorded to calculate the time that cells took for doubling their number. As expected, P10 MSCs took much longer time for doubling than P3 MSCs. Since the proliferation rate of P10 MSC is low, we further measured the cell number needed for generating a cell sheet in 24 hours. The results clearly showed a double amount of aged P10 MSCs is needed for generation of monolayer cell sheets when compared to P3 MSCs.

To determine whether aged MSCs could be used for bone tissue regeneration, we next transplanted both young P3 and aged P10 MSC sheets into a femoral allograft mouse model and compared them to allograft alone without MSC sheets. It has been reported that MSCs have negligible immunogenicity and the capacity for immune suppression. MSCs express low levels of major histocompatibility complex (MHC) class I molecules but lack expression of MHC class II molecules and the co-stimulary molecules CD80, CD86, and CD40^18, 19^. In addition, MSCs inhibit dendritic cells^20^, T cells^21^, B cells^22^, natural killer cells^23^, and macrophages^24^. Based on these facts, we decided to use wild type mouse for in *vivo* MSC transplantation. Since MSCs are immunoprivileged, we only performed gross observations to confirm the overall safety of human MSC sheet transplantation. As respiratory rate is an acute toxicity parameter for excessive inflammatory cytokines (such as tumor necrosis factor alpha [TNFα], interleukin [IL]-1α and IL-1β) aggregate in the microvasculature of the lungs that may cause cardiac and pulmonary distress^25^, we monitored mouse respiratory rate before and after surgery. Consistent with previous studies, there were no significant differences between allograft alone and MSC sheet groups regarding evaluation of respiratory distress upon human MSC transplantation. Gross observation analysis further showed that no significant difference was noticed in the three groups regarding the animal activity, skin color change, or weight loss, demonstrating the safety of transplantation of human MSCs into a mouse.

To further determine the therapeutic effect of aged P10 MSC sheets for bone tissue regeneration, a pre-clinic mouse model with a critical-sized bone defect was used in this study. Our data showed an elevated early soft callus formation in P10 MSC groups that is comparable to P3 MSCs at 3 weeks after surgery when compared to allograft alone groups. However, significantly less bony callus was observed surrounding P10 MSC sheet-wrapped allografts when compared with P3 MSC groups at 6 weeks post-surgery, indicating a delayed bony callus formation in P10 MSC sheet groups. Comparative studies have indicated that chondroid tissue ossification is a dynamic process marked by chondro-osteoclast mediated hypertrophic chondrocytes removal, type II collagen degradation and osteoblast-mediated type I collagen formation^26^. To further look at the possible mechanism responsible for the difference between P10 and P3 MSC sheet-mediated soft callus mineralization, we quantified the chondro-osteoclast positive area in the newly formed callus by TRAP staining. Our data clearly showed that osteoclast activity in the P3 MSC group was significantly higher than in P10 MSC groups at 6 weeks post-surgery, but not at 3 weeks. This increased chondro-osteoclast activity leads to accelerated removal of chondriod callus in P3 MSC group. In contrast, less numbers of chodro-osteoclasts in P10 MSC groups delayed the chondriod callus resorption and subsequent callus remodeling by showing more significantly chondriod callus formed in the aged MSC sheet groups. These data indicate the accelerated callus calcification and remodeling in P3 MSCs may be largely due to more osteoclasts in newly formed callus. Since this current study cannot demonstrate long-term survival of the MSC *in vivo*, more research needs to be performed to determine the cell autonomous and non-autonomous effects in MSC sheet-mediated chondro-osteclast formation during callus mineralization.

In summary, our study provides some insights on the differences between early-passaged young P3 MSC sheets and later-passaged aged P10 MSC sheets regarding the biological properties and chondro-osteogenic ability. Compared to young MSC sheets, aged MSC sheets show an equal chondrogenic effect and a reduced callus calcification. Further studies to enhance osteogenic ability of aged MSC sheets would be beneficial for future clinical translation.

## Materials and Methods

### Ethics Statement and Donor Data

Human bone marrow aspirate was harvested from the iliac crest of donors (n = 6), who were patients at University of Rochester Medical Center, had spine fusion surgery using their own bone marrow aspirate for MSC isolation. The mean age of the donors was 55 years (range 45–70). Donors included four males and two females with no history or evidence of genetic disease or malignancy. The Ethics Committee of University of Rochester approved the use of patient’s tissue material for this study (RSRB00048032). Written informed consent was obtained from all donors, and no identifying information will be recorded in this study. All methods for human experiments were performed in accordance with relevant guidelines and regulations.

### Human MSC isolation and culture

The method for isolation of human MSC was based on red blood cell (RBC) lysis with ammonium chloride^27, 28^. Briefly, 8 ml bone marrow aspirates from each patient were combined with 0.5 ml heparin anticoagulant (Becton Dickinson, San Jose, CA, USA; BD) in a syringe and delivered to our lab. After incubation in RBC lysis buffer for 10 min, total bone marrow cells were seeded at 5.0 × 10^6^ cells/cm^2^ in alpha-minimum essential medium (α-MEM) supplemented with 15% FBS, 2 mM L-glutamine, 0.5% antibiotic/antimycotic solution (all from Gibco-BRL, Life Technologies) for 3 days. Newly formed cell colonies were washed 3–5 times with 1XPBS and incubated at 37 °C in a humidified 95% air and 5% CO_2_ atmosphere, cultured up to 80% confluence, and then trypsinized (trypsin-EDTA solution, Gibco-BRL), centrifuged, and re-plated at a density of 20,000 cells/cm^2^ as P1 MSCs for subsequent expansion. After expansion for 2 passages, some of the P3 cells were harvested for flow cytometry analysis. The remaining P3 cells were divided into 2 populations for cell sheet formation as P3 MSCs and for continued passage until harvested as P10 MSCs for cell sheet culture. The MSCs from the patient with the highest expression of stromal markers were used to generate cell sheets in this study, and long-term cultures were always passaged at the same density of about 70–80% confluence. *In vitro* cell growth was monitored by cell number count and cumulative population doubling time (DT) calculation. DT was examined using the formula: (t − t0) ∙ log2/log (N − N0), where t − t0 is culture time in hours, N is the number of harvested cells, and N0 is the number of cells in the initial seeding^29^.

### Flow cytometry analysis

MSC phenotype was evaluated using P3 and P10 MSCs by the expression of CD34-APC (BD Biosciences), CD90–PE (BD Biosciences), CD73-PE (BD Biosciences) and CD105-FITC (R&D Systems, Minneapolis, MN). As previously described^30^, cells were incubated for 30 min at room temperature with antibodies, and flow cytometry was performed on an LSR-II flow cytometer (Beckton Dickson). Isotype matched controls were used to set the electronic gates on the flow cytometer. The data were analyzed using FlowJo software (Tree Star).

### Mouse study design

Animal use in this study was approved by Animal care and use committee at Louisiana State University Health Sciences Center. Allogeneic bone grafts were obtained from mice of the 129/J strain for implantation into C57BL/6 J mice. The Louisiana State University Committee of Animal care and use approved all animal surgery procedures (protocol#: P-15-005). Experiments were designed to include 12 male mice per group at different time points. Host mice carrying allografts were randomly and equally assigned to 3 groups: control (allograft alone), P3 MSC sheet/allograft and P10 MSC sheet/allograft groups. The sample size (n = 6) for Micro-CT and biomechanical testing was determined by power analysis based on our previous experiment data^5^. All methods for animal experiments were performed in accordance with relevant guidelines and regulations.

### Generation of MSC sheets

MSCs from P3 and P10 cultures were re-seeded at 200, 400, and 800 cells/mm^2^ on thermo-responsive 6-well culture plates with UpCell surface (Thermo Scientific, Cat. 174901) for cell sheet formation. After 24-hour culture, the cell sheet culture was monitored by Giemsa staining as previously reported^31^. The selected cell seeding density was used to generate a monolayer cell sheet (100% confluence) in 24 hours for subsequent *in vivo* implantation. Total RNA was extracted from both P3 and P10 MSC sheet cultures, Real time-PCR was performed as we previously described^30^ for ECM related genes: fibronectin, integrin β1, and type 1 collagen.

### Devitalization of bone allografts

Eight-week-old male 129/J mice were used for donation of devitalized allografts. Mice were euthanized, and a 4mm mid-diaphyseal segment was removed from each femur by osteotomy using a rotary Dremel with custom circular diamond blades. Allograft segments were flushed of the bone marrow using 25-gauge needles, the periosteal tissues were manually stripped, and the bone grafts were washed repeatedly in 70% ethanol for at least 4 hours. The allografts were then stored in 100% ethanol at −80 °C for at least 7 days to complete the devitalization process.

### Wrapping of MSC sheets on allografts

Following MSC sheet formation, cell sheets were wrapped onto devitalized allografts as described previously^5^. The cultured MSC sheets were covered by a cell transfer membrane (Thermo Scientific, Cat. 1749016) and kept at 25 °C for 10 minutes. After the cell layer adhered to the membrane, it was detached carefully from the thermo-responsive culture plate. The cell sheet and membrane were then placed in a new, larger dish with the cell layer facing up; next, the devitalized allografts were placed on top of the cell sheets, wrapped in one layer of cell sheet and incubated for an additional 30 minutes at 37 °C in fresh media to release the membrane. After carefully removing the membrane from the cell sheet, the MSC sheet wrapped allografts were kept at 37 °C for allograft transplantation surgery.

### Surgical reconstruction of the mouse femoral defects

Eight-week-old C57BL/6J mice were anesthetized via intraperitoneal injection with a combination of ketamine (60 mg/kg body weight) and xylazine (4 mg/kg body weight). A 7–8 mm incision was made, and the midshaft femur was exposed using blunt dissection of muscles. A 4-mm mid-diaphyseal segment was removed from the femur by osteotomizing the bone using the Dremel tool. A 4-mm bone graft with/without MSC sheets was then inserted into the segmental defect and stabilized using a 26-gauge metal pin placed through the intramedullary marrow cavity. The incisions were closed using silk sutures. Graft healing was followed radiographically using a Faxitron X-ray system (Faxitron X-ray Corporation, Wheeling, IL). Mice were sacrificed at 3 and 6 weeks post-surgery, and samples processed for further analysis.

### Safety evaluation

Safety parameters were evaluated, including respiration rates, activity, posture and skin color change caused by inflammation in the suture area before and 3 days after surgery. Weight loss was also monitored once a week for up to 6 weeks.

### Micro-CT bone imaging analyses

Some of the reconstructed femurs from week 3 and week 6 (n = 6) were imaged after careful dissection and removal of the intramedullary pin using a Micro-CT system (VivaCT 40, Scanco Medical). The femurs were scanned using a high-resolution (10.5 microns) x-ray with energy settings of 55 kVp and 145 µA. Quantification of bone volume (BV) and total volume(TV) of callus was performed as previously described using the Scanco analysis software^5^.

### Histological evaluation of grafted femurs

The femoral samples (n = 6) to be used for Micro-CT analyses were then fixed in neutral buffered formalin and processed in paraffin. Paraffin-embedded samples were sectioned at 5 μm and stained with Alcian Blue/Hematoxylin/Orange-G (AB/H/OG) to determine the contributions of cartilage, bone, and fibrotic tissue during the repair process using OsteoMeasure (TM) software^5^. Tartrate-resistant acid phosphatase (TRAP) staining was further performed in paraffin sections using kit (387 A) from sigma-aldrich. Positive area was quantified by ImageJ software.

### Biomechanical testing

Specimens (n = 6) from 6 weeks were harvested and moistened with saline before biomechanical testing. The ends of the femurs were cemented into square aluminum tube holders using polymethylmethacrylate (PMMA) to ensure axial alignment and to maintain a gage length of 7–8 mm, allowing a length of at least 3mm to be potted at each end. Specimens were mounted on an EnduraTec TestBench™ system (200 N.mm torque cell; Bose Corporation) and tested in torsion until failure. The torque data were plotted against the rotational deformation (normalized by the gage length and expressed as rad/mm) to determine the maximal torque and torsional rigidity as we reported previously^5^.

### Statistical analysis

The above experiments were repeated at least three times independently. All data are presented as mean ± standard deviation (SD). Statistical significance among the groups was assessed using one-way analysis of variance (ANOVA). The level of significance was *P* < 0.05.
